# Supercritical Carbon Dioxide Isolation of Cellulose Nanofibre and Enhancement Properties in Biopolymer Composites

**DOI:** 10.3390/molecules26175276

**Published:** 2021-08-31

**Authors:** Olaiya N. G., Abdul Khalil H. P. S., Salah M. El-Bahy, Mohd Rafatullah, Che K. Abdullah, Zeinhom M. El-Bahy, Olaiya F. Grace

**Affiliations:** 1 School of Industrial Technology, Universiti Sains Malaysia, George Town 11800, Penang, Malaysia; ck_abdullah@usm.my (C.K.A.); phunmieoseyemi@gmail.com (O.F.G.); 2 Department of Industrial and Production Engineering, Federal University of Technology, Akure PMB 704, Ondo State, Nigeria; 3 Department of Chemistry, Turabah University College, Taif University, P.O. Box 11099, Taif 21944, Saudi Arabia; s.elbahy@tu.edu.sa; 4 Department of Chemistry, Faculty of Science, Al-Azhar University, Nasr City, Cairo 11884, Egypt; zeinelbahy@azhar.edu.eg

**Keywords:** environmental, sustainability, crystallinity, supercritical, reinforcement, bionanocomposite

## Abstract

The physical properties, such as the fibre dimension and crystallinity, of cellulose nanofibre (CNF) are significant to its functional reinforcement ability in composites. This study used supercritical carbon dioxide as a fibre bundle defibrillation pretreatment for the isolation of CNF from bamboo, in order to enhance its physical properties. The isolated CNF was characterised through zeta potential, TEM, XRD, and FT-IR analysis. Commercial CNF was used as a reference to evaluate the effectiveness of the method. The physical, mechanical, thermal, and wettability properties of the bamboo and commercial CNF-reinforced PLA/chitin were also analysed. The TEM and FT-IR results showed the successful isolation of CNF from bamboo using this method, with good colloidal stability shown by the zeta potential results. The properties of the isolated bamboo CNF were similar to the commercial type. However, the fibre diameter distribution and the crystallinity index significantly differed between the bamboo and the commercial CNF. The bamboo CNF had a smaller fibre size and a higher crystallinity index than the commercial CNF. The results from the CNF-reinforced biocomposite showed that the physical, mechanical, thermal, and wettability properties were significantly different due to the variations in their fibre sizes and crystallinity indices. The properties of bamboo CNF biocomposites were significantly better than those of commercial CNF biocomposites. This indicates that the physical properties (fibre size and crystallinity) of an isolated CNF significantly affect its reinforcement ability in biocomposites. The physical properties of isolated CNFs are partly dependent on their source and production method, among other factors. These composites can be used for various industrial applications, including packaging.

## 1. Introduction

Polymers are broadly classified as either synthetic or natural [1]. Natural polymers are often called biodegradable polymers, or simply biopolymers. Biodegradable polymers have been used as replacements for synthetic ones because of their biodegradable properties [1]. The pollution caused by synthetic polymers has resulted in global pollution, both inland and in marine habitats [2]. PLA is a naturally-sourced polymer with good biodegradability, biocompatibility, and nontoxic properties. PLA has similar properties to many synthetic polymers, which is why it is considered for their replacement [3]. PLA is produced for industrial use from the polymerisation of lactic acid [4]. It is one of the most economical polymer preparation processes. PLA has been reported as having a wider range of applications compared with other biopolymers. It can be modified into different forms, of low and high molecular weight. Much research has been conducted on the modification of PLA for industrial applications [5]. PLA surfaces have been treated with gamma-ray, coupling agents, and chemicals to enhance their properties. PLA is nontoxic, which makes it suitable for food packaging.

PLA has excellent water resistance, which makes it suitable for fluid packaging. Furthermore, the degradation properties of PLA have been studied [6]. The biodegradation of PLA consists of several processes, such as hydrolysis, microbial, and enzymatic. A previous report showed that the degradation process of PLA is prolonged, since it has resistance to microbial attack [6]. PLA microbial degradation does not start until its surface is hydrolysed. This often results in slow degradation on soil burial test analysis, as reported. However, the mechanical strength of PLA is too low to sustain its several potential applications. PLA has mainly been reinforced with nanoparticles to enhance its mechanical strength [4,7]. Nanocellulose is one of the major biopolymers used for PLA reinforcement.

The discovery of nano-sized material has resulted in more research on the reinforcement of biopolymers for packaging applications [8,9,10]. Bamboo, which has been classified within a special type of grass family, called Gramineae, offers excellent strength because of its hollow trunk (culms) [11]. Bamboo trunks are similar in strength to wood; however, bamboos have been classified as grasses because their trunk shoots from the ground, a characteristic unique to the grass family [12]. Despite its hollow trunk, bamboo has similar characteristics to grass plants. The difference between grass and bamboo has been found in the distinctive organisation within the internal structure of bamboo leaves. Bamboo is rich in cellulose-like wood but consists of several complex branches. The properties of isolated nanocellulose have been reported as being likely to be dependent on its source and production method. However, this has not been established by previous research. Therefore, in this study, nanocellulose was isolated from two different natural sources [13].

Chitin is available from crustacea and arthropods, as well as from fungi and yeast. However, its main source is crab and shrimps’ shell waste [14]. Chitin has a structure similar to cellulose, except for the presence of the amine functional group. Chitin is biodegradable, biocompatible, and nontoxic. Chitin is a polysaccharide with a nitrogen-containing functional group called amine [14]. It is usually synthesised chemically from *N*-acetyl-D-glucosamine (to be precise, 2-(acetylamino)-2-deoxy-D-glucose). Like cellulose, this unit forms a covalent β-(1→4)-linkage in the glucose units [15]. Therefore, chitin is often described as cellulose with hydroxyl and amine groups. The hydroxide functional group allows hydrogen bonding with adjacent polymers with a hydrophilic nature, such as cellulose. Chitin has good miscibility with cellulose and PLA, and its blend has been researched [16]. A previous study of PLA/CNF reported agglomeration and that chitin could be used as a compatibilizer between PLA and CNF, since it mixes very well with them [17,18].

Therefore, CNF was isolated from bamboo in this study through supercritical carbon dioxide explosion defibrillation pre-treatment, acid hydrolysis, and high-pressure homogenisation. The isolated CNF was characterised using x-ray diffraction (XRD), zeta potential, Fourier transformed infrared spectroscopy (FTIR), and transmission electron microscopy (TEM). The properties of the isolated bamboo CNF were standardised using the commercial type. Supercritical CO_2_ has been used in the fluid extraction [19] and drying of CNF [20,21]. The incorporation of supercritical CO_2_ as a defibrillation pretreatment in the isolation of CNF has not been conclusively studied. Furthermore, the evaluation of the technique by comparing it with the conventional method has not been reported. The reinforcement properties of the bamboo CNF (B-CNF) and the commercial CNF (C-CNF) in PLA/chitin biocomposites were studied through physical, mechanical, thermal, and wettability analysis.

## 2. Results

### 2.1. The Properties of Bamboo and Commercial Cellulose Nanofibres

**Figure 1:**
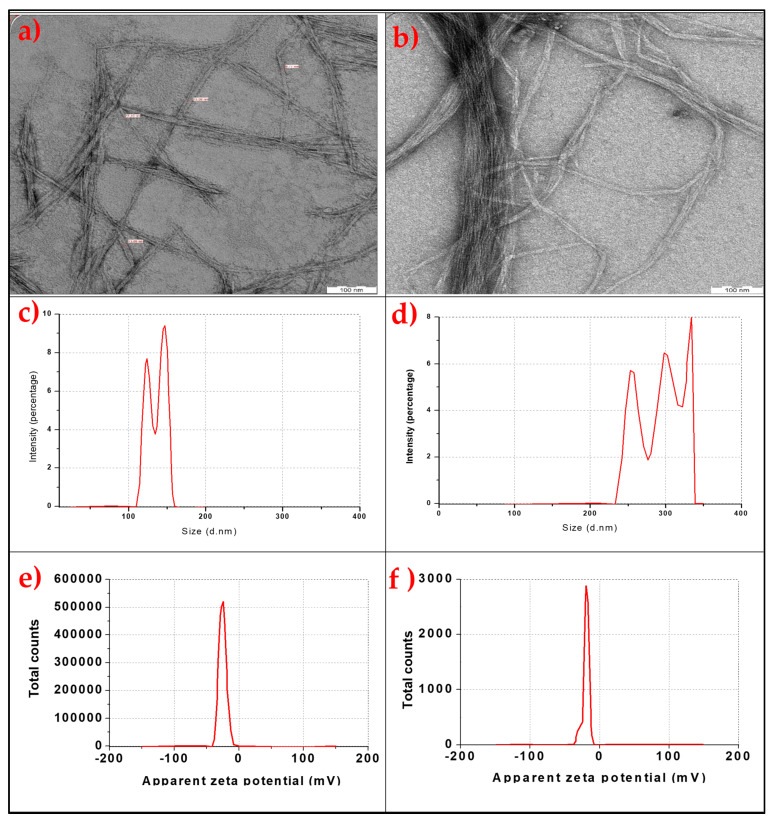
TEM images of (**a**) bamboo CNF; (**b**) commercial CNF, particle size analysis; (**c**) bamboo CNF; (**d**) commercial CNF, zeta potential analysis of (**e**) bamboo CNF; and (**f**) commercial CNF.

The results of the TEM, particle size analysis, and zeta potential value of the bamboo and commercial CNF are presented in Figure 1a–f.

The micrograph images of the transmission electron microscopy (TEM) analysis for the bamboo and the commercial CNF are shown in Figure 1a,b, respectively. The bamboo CNF and commercial CNF showed a similar TEM morphological pattern of irregular rod shape with network fibres. The bamboo CNF showed a well-dispersed, rod-like network with no fibre bundles, while the commercial CNF still had some fibre bundles in its TEM image. The bamboo CNF exhibited fibrous morphology, probably due to its precursor material (parent fibre) [22,23]. At the same time, the commercial CNF showed no regular fibre length, which can also be attributed to its source (cotton) [24]. Commercial CNF (C-CNF) derived from cotton also showed irregular and rough surfaces with some aggregate particles [24]. Bamboo CNF had a smaller fibre size than commercial CNF, as analysed using particle size analysis. The fibre diameter size is one factor that determines the reinforcement ability of CNF in composites [25]. Slight morphological differences between bamboo CNF and commercial CNF’s physical properties could probably be due to differences in the source of their raw materials and their production methods [26]. The potential value of isolated CNF was assessed in order to measure its stability [27]. The zeta potential analyses of bamboo CNF and commercial CNF are presented in Figure 1e,f, respectively. The potential of each type of CNF had values ranging between 0 and 50 V. Previous reports on zeta potential analysis show that a potential above 20 V is considered stable in the colloidal fluid [28,29]. Since both CNF were higher than 20 V, the high voltage indicated stable particle materials. These results confirmed that the isolated CNF could not be modified back to its raw material or have its properties changed, even at high voltage. This means that the isolation process produced a stable cellulose nanofibre [29].

**Figure 2:**
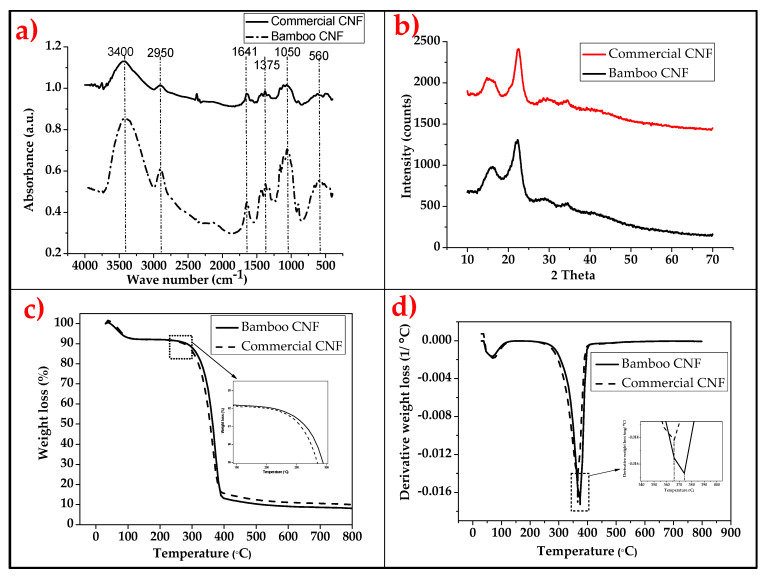
Results obtained from bamboo and commercial CNFs by (**a**) FT-IR; (**b**) XRD; (**c**) TGA; and (**d**) DTG.

The results of the FT-IR, XRD, and TGA-DTG analyses of the bamboo and the commercial CNF are presented in Figure 2a–d.

Both FT-IR bands (Figure 2a) showed a wavenumber band of 3600–3200 cm^−1^, suggesting the stretching of hydroxide, corresponding to hydrogen bonds in the structure (-H of the –OH group). The wavenumber band at 2969 cm^−1^ and 2902 cm^−1^ corresponds to CH-stretching vibrations. The wavenumber at 1640 and 1643 cm^−1^ shows typical bending of water molecules due to the strong interaction between cellulose and water. Furthermore, the band at 1375 cm^−1^ represents the vibration bending and wagging of CH_2_ and CH. The band at 1284 cm^−1^ shows C-O-C stretching from the β-1, 4-glycosidic linkage in cellulosic material, while the 1058 cm^−1^ wave band indicates –CH_2_-O-CH_2_ Pyrenees ring stretching vibration [30]. The wavenumber band at 810 cm^−1^ and 563 cm^−1^ represents C-H out of plane stretching in cellulose due to β-linkage. The absence of a wave number band at around 1512–1562 cm^−1^ and 1700–1740 cm^−1^ indicates successfully eliminated lignin and hemicellulose [31]. This confirms that the CNF produced was of high quality. These bonds are a typical indication of isolated cellulose nanofibre, in line with previous reports by Atiqah et al. [32]. The difference between the bamboo CNF and the commercial one is in the intensity of the representative absorbance band for each bond identified. The difference in bands is more significantly shown in the hydrogen bonding between 3600–3200 cm^−1^, which is higher in bamboo CNF than in commercial CNF [33]. An absent peak at around 1512–1562 cm^−1^ (lignin) and 1700–1740 cm^−1^ (hemicellulose) indicates CNF production was high in purity [34].

The results of the XRD analysis of the bamboo CNF and the commercial CNF is presented in Figure 2b. The results showed two significant peaks for both CNF at 2 theta equals 15° and 22.5°. However, the peak intensity of the bamboo was observed to be greater than that of the commercial CNF, which is an indication of a possible difference between their crystallinity indices. The XRD analysis was used to analyse the crystallinity indices of the two types of CNF. This is important because it has a significant effect on their reinforcement ability [24]. The XRD crystallinity of bamboo CNF was 75.68% greater than that of commercial CNF, which was 70.67%. The higher crystallinity index means a higher reinforcement effect in composite materials. The major crystalline peak observed in this study is similar to those previously reported. This shows that the isolated CNF represented the cellulose structure, and that the crystal integrity of CNF was affected by the preparation method of the bamboo CNF [35]. Higher crystallinity means greater efficiency in achieving a higher reinforcement effect in composite materials. The major crystalline peak for all samples, occurring at around 2θ = 22.5 (no doublet found), represents cellulose I structure, and suggests that crystal integrity has been maintained [36].

The thermogravimetry analysis of the bamboo CNF and the commercial CNF is shown in Figure 2c,d. Both CNFs showed a similar TGA curve (Figure 2c), with initial water evaporation below 100 °C. The onset temperature of the commercial CNF was lower than that of the bamboo, at 215 °C and 208 °C, respectively. The difference was probably due to the source of the CNF. The degradation percentage of both CNFs showed similar descending values. Furthermore, the DTG curve (Figure 2d) showed a significant peak difference between the bamboo and commercial CNFs at 375 °C and 366 °C, respectively. The DTG curve corroborates the observation that the differences between the TGA onset temperatures suggested a difference in the thermal properties of the CNFs, which may have been due to their sources [37,38].

### 2.2. The Physical Properties of Bamboo and Commercial CNF-Reinforced PLA/Chitin Bionanocomposites

**Figure 3:**
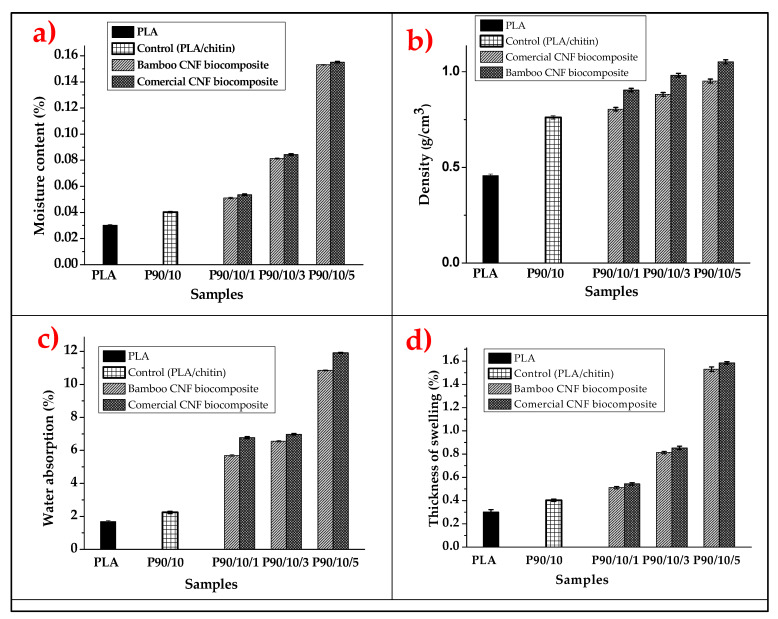
(**a**) Moisture content; (**b**) density; (**c**) water absorption; (**d**) swelling thickness properties of bamboo and commercial CNF-reinforced PLA/chitin.

The physical properties of the biocomposite were evaluated using moisture content, water absorption, thickness swelling, and density evaluation, as presented in Figure 3a–d.

The moisture content for neat PLA, PLA/chitin biocomposite, and PLA/chitin/CNF biocomposite is plotted in Figure 3a. The graph shows that the samples’ moisture content varied between 0.02% to 0.17%. The neat PLA was observed to have the lowest moisture content. In comparison, the biocomposite with 5% cellulose nanofibre had the highest (0.2%) moisture content. The moisture content increased with the addition of chitin and cellulose nanofibre compared with that of neat PLA. The lowered value of the neat PLA’s moisture content was probably due to the hydrophobic nature of PLA. The addition of chitin to the neat PLA slightly increased its moisture content because of increased surface area and the hydroxyl functional group present in chitin. However, the addition of CNF had a more significant effect on the biocomposite’s moisture content because of the hydrophilic nature of CNF [20]. Cellulose nanofibre has a higher water content, and its addition to PLA/chitin increases its moisture content. The possible internal bonding between the three polymeric materials generally affected the composite water content of the composite because some hydroxy groups must have been used for bonding. This may have been responsible for the low water absorption of the biocomposite.

The difference in the moisture content between PLA/chitin and PLA/chitin/CNF is explained by the introduction of more hydroxyl groups with increased CNF content. A previous study on the moisture content of PLA/chitin and PLA/CNF showed a similar trend. The moisture content from the PLA/chitin composite study by Nasrin et al. [39] showed an increase with a higher percentage of chitin. Furthermore, similar studies confirmed an increase in the PLA/chitin composite’s moisture content compared with the neat PLA [40,41]. They explained that it was probably due to space between the PLA and the chitin’s molecular arrangements at a higher percentage. Studies on PLA/CNF showed CNF added to the moisture content because of its water-containing cellulose ability [42]. Neat PLA had the lowest moisture content. The PLA-chitin (P9010) composite moisture content increase compares to neat PLA. 5% CNF fillers, which are highest among PLA-chitin-CNF biocomposites. The bamboo CNF (B-CNF) biocomposite has a lower moisture content value than C-CNF biocomposites at the same loadings. The difference between the moisture content values is probably due to the higher crystallinity index of B-CNF, which prevents water absorption compared with the C-CNF. The difference between the indices of B-CNF composite and C–CNF composite is due to the preparation or the precursors of CNF.

The neat PLA’s density values, P9010, P90101, P90103, and P90105, are plotted in Figure 3b. The neat PLA had the lowest density, while the biocomposite with 5% CNF had the highest. Similarly, the addition of chitin to the matrix enhanced the PLA density. The results show that the loading of CNF increased the density of the material. This means that the nanofibre spread across the material’s internal structure, filling the voids in the structure. The increase in density value with the addition of CNF is largely due to the nanosize of the fibre [22]. Nanoparticles probably filled up possible voids or hollow spaces in the bio-composite bundles, resulting in compacted material. Mathematically, density is calculated from mass or weight per unit of volume. This means that the biocomposite mass increases with the addition of CNF while still occupying the same volume, resulting in an increased density value [43]. However, the density of the commercial fibre was slightly lower than that of bamboo, which may have been due to the difference in their crystallinity and fibre sizes. The bamboo CNF had a smaller fibre size, contributing to its ability to fill smaller openings than the commercial type.

The PLA-Chitin biocomposite had the lowest water absorption, as show in Figure 3c. Furthermore, the 5% CNF loading led to the highest water absorption among the PLA-chitin-CNF biocomposites. The B-CNF composite comprised reduced water absorption properties compared to C-CNF biocomposites at the same loadings, probably due to the high crystallinity of the bamboo, which prevented water absorption. The biocomposite’s water absorption properties are significant because they determine the material’s environment and its dimensional stability. A biocomposite with high water absorption may cause a weakening of its internal bonding, reducing its mechanical strength [44]. Fibre loading is one of the parameters that significantly affect the water absorption properties of biocomposite [45]. The water absorption percentage was observed to increase significantly when compared with the neat PLA. The water absorption value increased with the addition of chitin and CNF. The water absorption values of neat PLA and PLA/chitin seem to have been close, despite the addition of 10% of chitin, but they were significantly higher with CNF [39]. This shows that the addition of CNF has a higher effect on water absorption properties than chitin [46]. This is probably due to the hydrophilic nature of CNF because of the crowded hydroxyl presence in its structure.

Chitin, on the other hand, is considered hydrophobic because it is not soluble in water. This difference in the nature of chitin and CNF has a more significant impact on the water absorption properties of the resulting PLA/chitin/CNF [47]. The result of the water absorption value showed that the properties depend on the fibre reinforcement’s nature and the quantity of the reinforcement in the PLA [18]. This observation is similar to those made in previous studies on the water absorption properties of PLA containing chitin or CNFs [48]. In these studies, it was reported that the nature of the filler or fibre reinforcement had a significant effect on the water absorption properties. Furthermore, because chitin is hydrophobic, it provides more water absorption stability to the biocomposite than the CNF. The increase in CNFs in the biocomposite results in neat PLA’s ability to form hydrogen bonds with water [49]. This resulted in more water absorption of the biocomposite with the addition of the CNF. However, the biocomposite water absorption properties showed characteristics of hydrophobic material [50]

The thickness swelling of neat PLA, PLA/chitin, and PLA/chitin/CNF biocomposites after immersion in water for 24 h is plotted in Figure 3d. The thickness of the PLA/chitin and PLA/chitin/CNF increased compared with that of the neat PLA. Neat PLA has a lower swelling thickness [51]. The PLA-chitin composite increase compares to that of neat PLA. The 5% CNF loading was the highest among the PLA-chitin-CNF biocomposites. However, B-CNF composites have reduced swelling values compared to C-CNF biocomposites at the same loadings [47]. The B-CNF biocomposite’s lower value than C-CNF for thickness was probably due to the high crystallinity of the bamboo, which prevents water absorption, which is a ripple effect from their source [52]. The thickness trend is similar to that of water absorption. The swelling thickness of the neat PLA did not significantly change due to its hydrophobic nature. The thickness of the PLA’s swelling increased with the addition of chitin, as observed in the P9010 biocomposite. This was probably due to the space created by the presence of chitin particles in the PLA matrix. Furthermore, the swelling thickness of PLA/chitin/CNF (i.e., P90101, P90103, and P90105) increased with the addition of CNF, which can be explained by the hydrophilic nature of CNF. Generally, swelling thickness observed in this study depended on the polymer mix’s nature and the porosity (the voids or space) between the polymer mix molecules [51]. The value of the percentage thickness of swelling was relatively low, which shows that the biocomposite has low porosity and is highly resistant to water [53].

### 2.3. The Mechanical Properties of Bamboo and Commercial CNF-Reinforced PLA/Chitin Bionanocomposites

**Figure 4:**
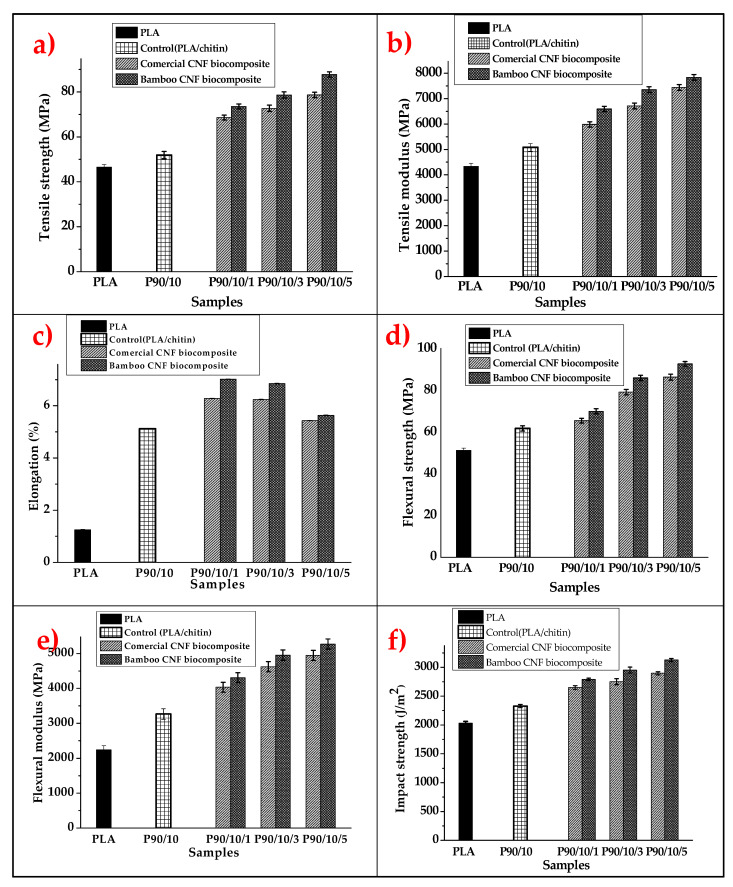
Comparative analysis of (**a**) tensile strength; (**b**) tensile modulus; (**c**) elongation; (**d**) flexural strength; (**e**) flexural modulus; and (**f**) impact strength properties of bamboo and commercial CNF-reinforced bionanocomposites.

The comparative mechanical strength analysis of the bionanocomposites was evaluated using tensile, flexural, and impact properties evaluation, as presented in Figure 4a–f.

The results of the comparative tensile properties of bamboo and commercial CNF reinforcement in PLA/chitin composite are presented in Figure 3. The PLA/chitin (P9010) biocomposite had the lowest tensile strength, of 51.06 MPa, while the 5% CNF loading had the highest tensile strength. The B-CNF biocomposites were observed with better reinforcement properties than the C-CNF biocomposites at the same CNF percentage loadings. The B-CNF biocomposites showed better tensile strength properties than the C-CNFs. The difference between the tensile strength values of the biocomposites was probably due to the high crystallinity (XRD) and lower fibre size (TEM) of the B-CNF compared to the C-CNF. The crystallinity index and fibre size of CNF were dependent on the method of isolation and precursor material.

Figure 4b shows the tensile modulus values from the comparative analysis between the B-CNF biocomposite and the C-CNF biocomposite at the same loading. The tensile modulus showed a similar trend to the tensile modulus value. The PLA/chitin (P9010) biocomposite had the lowest tensile modulus (5081 MPa) for all samples. The tensile modulus value for both the B-CNF and the C-CNF biocomposites increased with its percentage loading. The biocomposites with 5% CNF loading had the highest tensile modulus. However, the B-CNF biocomposites showed better reinforcement properties than the C-CNF composites at the same loading from the tensile modulus value. This shows that B-CNFs offer better tensile properties enhancement than C-CNFs in biocomposites, probably due to their high crystallinity (XRD) and lower fibre size (TEM). This factor is directly dependent on the method of preparation of CNFs and their material source. Previous studies on PLA/CNF confirm that cellulose nanofibre’s reinforcement ability is dependent on its crystallinity index and fibre size, among other factors. Yu et al. [54] studied the reinforcement effect of different cellulose nanofibre aspect ratios on polylactic acid. The resulting composite showed that its tensile modulus properties were significantly dependent on the fibre aspect ratio. Other studies on the tensile modulus of PLA/CNF biocomposite reported a similar trend [18].

Figure 4c presents the elongation of B-CNF and C-CNF biocomposites with PLA/chitin (P9010) as the control. The PLA/chitin (P9010) biocomposite had the lowest elongation (5.12%). The elongation value for all PLA/chitin/CNF biocomposites was higher than the control film, which shows that the brittle properties of PLA were reduced with chitin and CNF. However, 1% CNF loading had the highest elongation in this case, and the elongation value reduced with increased CNF. Furthermore, the B-CNF biocomposites’ elongation values were slightly higher than the C-CNF biocomposites for all the same loadings. The fact that B-CNF biocomposite had better elongation than C-CNF biocomposite was probably due to the higher stiffness enhancement resulting from its higher crystallinity index (XRD). This was also dependent on the physical properties of the isolated CNF.

The results on the flexural strength graph in Figure 4d show an increase of CNF loading. The neat PLA/chitin had the lowest, and the 5% loading had the highest. The flexural strength of the B-CNF set of samples was higher than those of C-CNF samples due to the differences between their crystallinity indices and fibre sizes. The source of the CNF and the preparation method play a significant role in the properties of the resulting biocomposite. The flexural strength increased from 61.88 MPa to 92.8 MPa for 5% B-CNF to 5% CNF loading, while C-CNF increased to 86.4 MPa for similar loading. The flexural strength of the biocomposite was probably due to its high crystallinity (XRD) and lower fibre size (TEM). The difference between the results of the B-CNF composite and the C-CNF composite was due to the preparation of the CNF and source of the CNF. CNF at different loadings increased the biocomposite’s surface area, contributing to stress distribution and making the film resistant to bending load. A previous investigation shows a similar trend towards an increase in flexural properties with the addition of CNF. However, little has been reported on the comparative properties of different sources of CNFs. A previous report on the effect of cellulose crystallinity on the flexural properties of biocomposite showed a significant variation between the thermomechanical properties of different sources of cellulose [55].

Figure 4e shows the flexural modulus of PLA/chitin, PLA/chitin/B-CNF, and PLA/chitin/C-CNF biocomposites. The modulus value increased with CNF loading, from 3263 MPa for P9010 to 5071 MPa for 5% B-CNF loading and 4946 MPa for 5% C-CNF loading. The PLA/chitin had the lowest flexural modulus, followed by PLA/chitin with 1% CNF(P90101). The biocomposite with 5% CNF had the highest flexural modulus of the two sources. However, the flexural modulus values for the B-CNF were higher than those of the C-CNF at the same loadings. Therefore, B-CNF composite can be regarded as a better flexural modulus enhancer of biocomposites than C-CNF, probably due to its higher crystallinity (XRD) and lower fibre size (TEM), depending on their method of preparation and source.

Figure 4f shows the comparative impact strength properties of B-CNF and C-CNF reinforced by the biocomposite. The PLA/chitin had the lowest impact strength. The graph’s PLA-Chitin (P9010) biocomposite impact strength was 2627 MPa, increasing with CNF loading. Furthermore, 5% CNF fibre reinforcement had the highest impact strength among the PLA-chitin-CNF biocomposites. However, the B-CNF-reinforced biocomposites had a higher impact strength reinforcement property than C-CNF composites at the same loadings. This shows that the biocomposite became more crystalline which in turn resulted in high impact strength. The difference in the results of the B-CNF composite and C-CNF composite was due to the method of preparation and source of the CNF. The result shows the significant impact of the fibre size and crystallinity of CNF on its reinforcement ability.

### 2.4. The Morphological Properties of Bamboo and Commercial CNF-Reinforced PLA/Chitin Bionanocomposites

**Figure 5:**
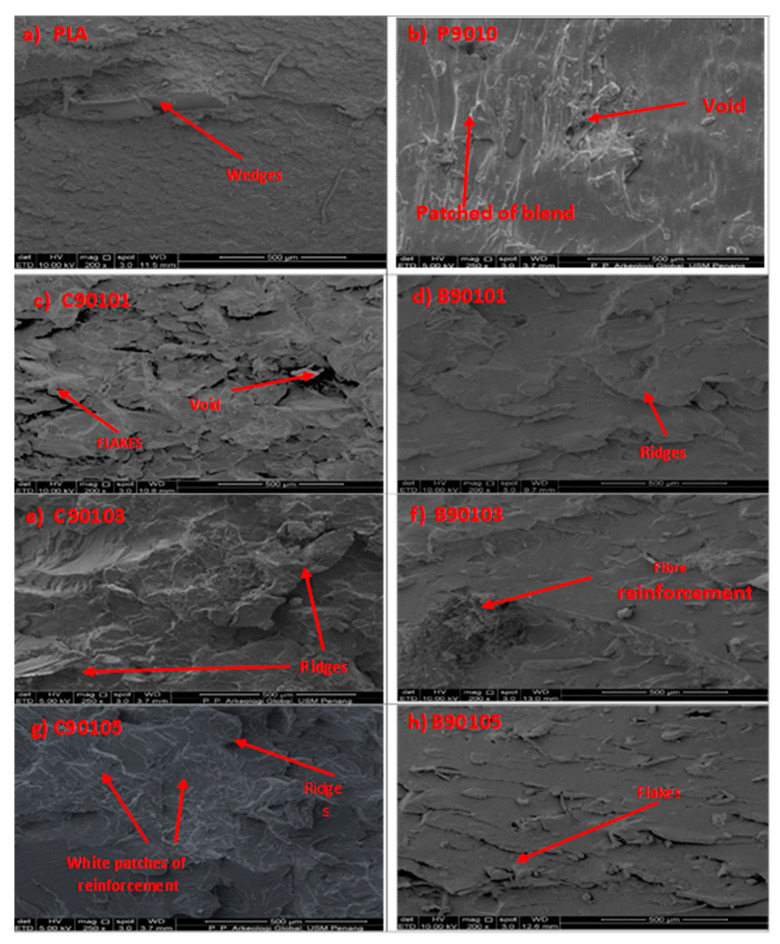
Comparative analysis of the morphological properties of bamboo and commercial CNF-reinforced biocomposites.

The comparative analysis of the morphological properties of the B-CNF- and C-CNF-reinforced biopolymer composites is shown in Figure 5. The fractured surface morphology micrograph of the neat PLA, the PLA/chitin (P9010), and the PLA/chitin/CNF for B-CNF (B9010, B90103, B90105) and C-CNF (C90101, C90103, C90105) biocomposites are shown in Figure 4a–f. The morphological images showed good miscibility between biopolymers with no segregation. The images showed increased roughness in the biocomposite morphology with the addition of CNF. This observation means that the biopolymer blends were thoroughly compacted together with no void. This was probably responsible for the high tensile strength and modulus values observed in the mechanical analysis. The neat PLA’s morphological changes when chitin and CNF were added can be clearly seen in SEM images. Wedges and flakes characterise the addition of chitin.

At 1% CNF there was no significant difference between the SEM images of the bamboo CNF and commercial CNF. However, at 3% and 5%, a network of white patches was observed in the fractured surface of the commercial CNF, while the bamboo CNF still had a uniform colouration. This network of patches in the SEM images probably showed signs of pure chitin or CNF, though these were insignificant as no void or cracks were observed. The network of CNF fibres was shown to increase in the images from 1% to 5%. Previous studies on PLA/CNF biocomposites reported the agglomeration of CNF in PLA due to the difference in their nature [18]: PLA is hydrophobic, and CNF is hydrophilic. The difference in the nature of PLA and CNF probably resulted in poor mechanical properties. The SEM images indicated that the neat PLA exhibited a smooth, homogeneous, and compact fractured surface. The SEM images were characterised by flakes, ridges, and rough surfaces with increasing fibre content. Rougher surfaces were observed in the 5% CNF composite, with white particles (probably CNF) embedded on the PLA/chitin matrix surfaces.

### 2.5. The Structural Properties of Bamboo and Commercial CNF-Reinforced PLA/Chitin Bionanocomposites

**Figure 6:**
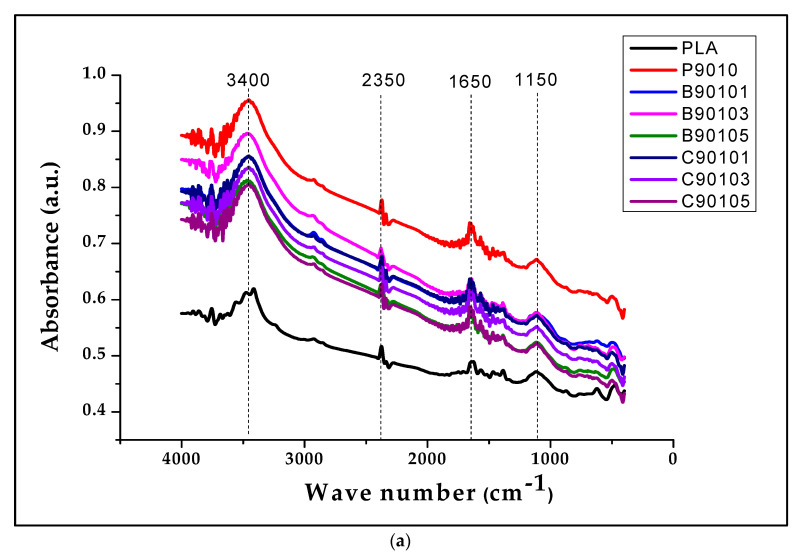

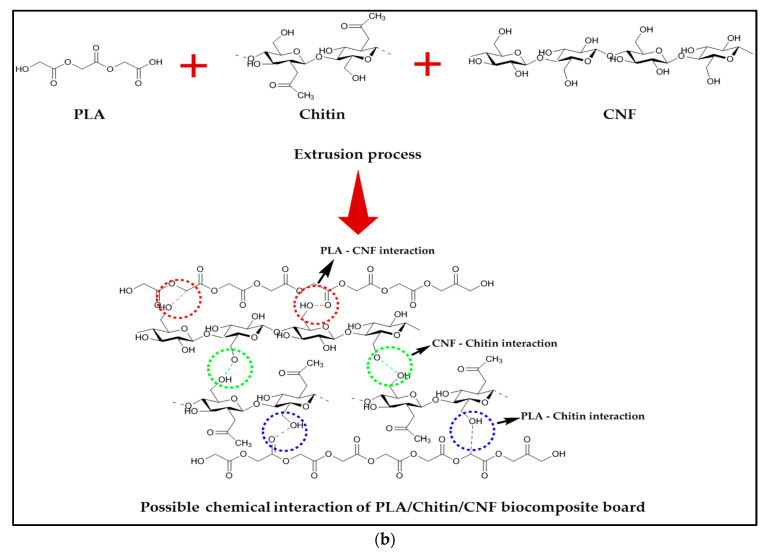
(**a**) Comparative analysis of the FT-IR properties of bamboo and commercial CNF-reinforced biocomposites; (**b**) Schematic chemical bonding in CNF-reinforced biocomposites using ChemDraw software.

**Figure 7:**
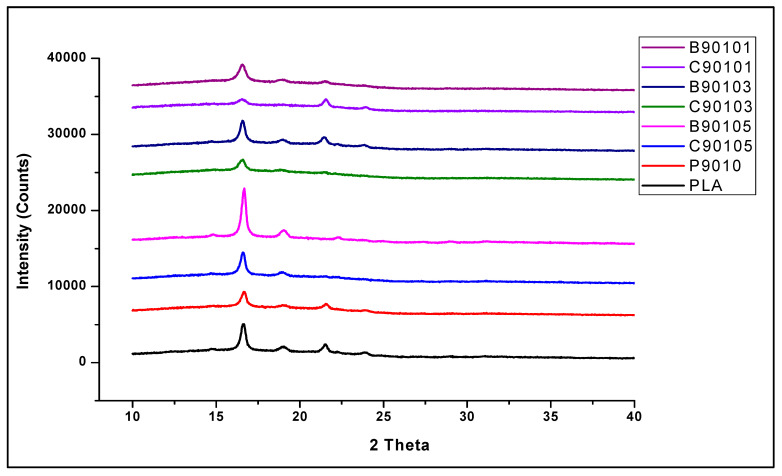
Comparative analysis of X-ray diffraction analyses of bamboo and commercial CNF-reinforced biocomposite.

The structural properties of the biocomposites were evaluated using FT-IR and XRD analysis, as presented in Figure 6 and Figure 7.

The FT-IR analysis of B-CNF- and C-CNF-reinforced biocomposites is presented in Figure 6a, and the schematic diagram of possible bonding is presented in Figure 6b. The results showed similar bands and stretches in the FTIR spectra. The band stretch between 3300 and 3600 cm^−1^, which can be attributed to OH, confirmed presence of hydrogen bonding in the biocomposite. The lower band stretches below 1500 cm^−1^ for all samples, can be attributed to C-H and C-C linkages common for all polysaccharides. The FT-IR spectra between 3400 and 3650 cm^−1^ were due to the OH band present in chitin and CNF. The band at 2950 cm^−1^ was attributed to C-H stretching vibrations, which are typically present in PLA, chitin, and CNF. The spectra band at 1654 cm^−1^, 1599 cm^−1^, and 1550 cm^−1^ was assigned to amide, like that present in chitin. However, peaks such as 1154 cm^−1^, 850 cm^−1^, and 650 cm^−1^ were not significantly observed in the biocomposites. In general, both bamboo CNF and commercial CNF had similar functional groups. This was expected because the chemical structure of CNF is not affected by its source and method of production; otherwise, the CNF isolated is not pure [37,56].

The results of the XRD analysis of PLA/chitin-, B-CNF-, and C-CNF-reinforced biocomposites is presented in Figure 7. The result shows peaks between 2θ = 16° to 22°, with the highest peak at 2θ = 16°. The neat PLA used in this study was semicrystalline, and these peaks are due to the crystalline parts. The remaining part of the graph, without any peaks in the amorphous region, shows that the biocomposite was more amorphous, as shown by the span of the non-peak region. The addition of chitin to neat PLA is shown with sample P9010, which reduced the XRD peak height compared to PLA/chitin/CNF [57]. However, as was reported in previous studies, the three peaks in the neat PLA were still maintained only to reduce the intensity [58]. Furthermore, similar peaks were observed throughout the biocomposite with B-CNF and C-CNF loading, with slightly higher peaks than the P9010 (control sample) [59].

The peaks were mostly similar in all the biocomposite regions, probably due to the high percentage of PLA present in the biocomposites. The B-CNF-reinforced biocomposite peaks had a higher peak intensity than the C-CNF-reinforced biocomposites with the same percentage loading. This was expected, since there is a significant difference between the crystallinity indices of these two types of CNF. The XRD crystallinity of the bamboo CNF was 75.68%, which is greater than that of the commercial CNF, 70.67% (Figure 2b). This is important because it has a significant effect on their reinforcement ability. A higher crystallinity index means a higher reinforcement effect in composite materials. Cellulose and polylactic acid have been reported to have similar crystalline peak regions in XRD analysis [60], and the peak is maintained or increased with the addition of CNF loading [61].

### 2.6. The Thermal Properties of Bamboo and Commercial CNF-Reinforced PLA/Chitin Bionanocomposites

**Figure 8:**
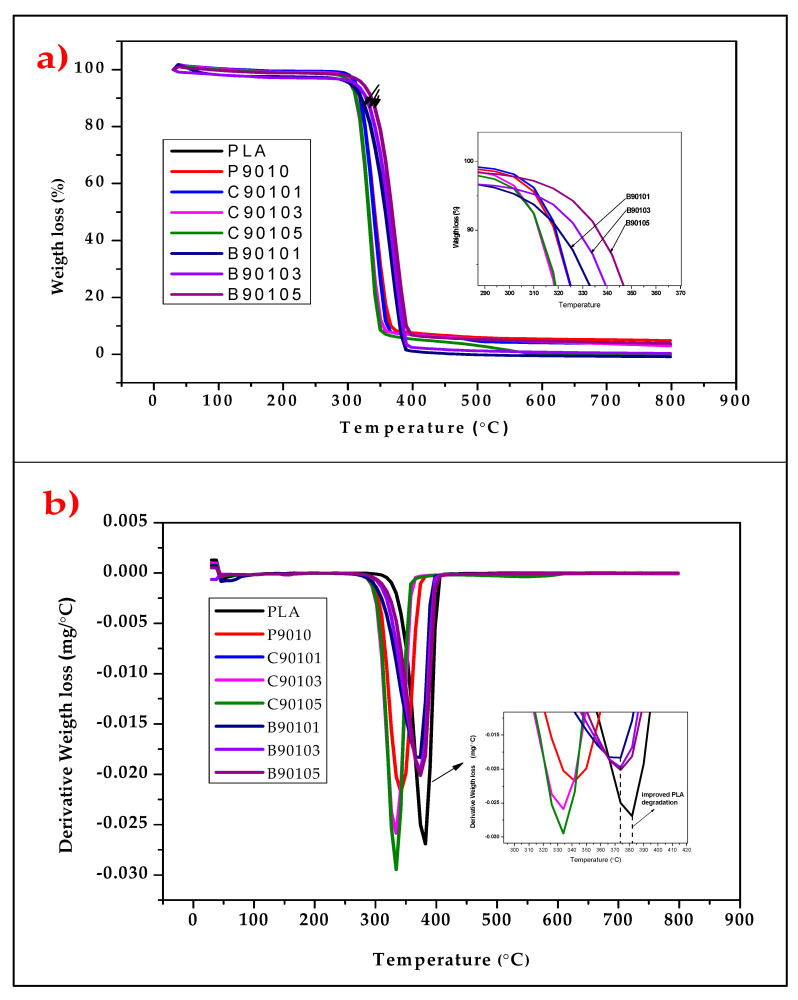
Comparative analysis of (**a**) TGA and (**b**) DTG properties of bamboo and commercial CNF-reinforced biocomposites.

The thermogravimetry analysis results ae shown in Figure 8. The Figure shows two degradation steps: initial and significant degradation. The onset temperature shows the beginning of the degradation process of the material content. The initial degradation occurred below 50–65 °C for the PLA/chitin-, B-CNF-, and C-CNF-reinforced biocomposites. The thermal degradation of the samples began with the initial step, which can be attributed to the evaporation of water and the volatile content (vaporisation of moisture) of the composites. The second degradation onset of PLA/chitin was ~285 °C. Thermal stability tends to improve with the addition of the two types of CNF fibres. The onset temperature of 5% CNF biocomposite was the highest. The second degradation shown on the graph occurred at a temperature between ~300 and 310 °C for the B-CNF biocomposites and between 290 and 300 °C for the C-CNF biocomposites. As was observed from this range of values, the B-CNF biocomposites had a higher onset temperature than the C-CNF biocomposites. The major decomposition peaks observed on the DTG graph (Figure 8b) show that the B-CNF biocomposites’ peak degradation temperature ranged from ~360 to 380 °C, which was greater than that of the C-CNF biocomposites, at ~340–354 °C peak temperature. These values probably show that B-CNF reinforced biocomposites have better thermal stability than C-CNF-reinforced biocomposites [62]. However, both biocomposites had percentage degradation values above 80% of the weight loss decomposition point of biocomposites [63]. PLA/chitin reinforced with B-CNF has a higher thermal stability than when it is reinforced with C-CNF, probably due to the difference between the crystallinity properties of the CNFs [64].

### 2.7. The Wettability Properties of Bamboo and Commercial CNF-Reinforced PLA/Chitin Bionanocomposites

The wettability properties of the bionanocomposite were evaluated using contact angle analysis. The results of the contact angle analysis, shown in Table 1, showed that the values were lower than 90°. The contact angle values ranged from 78.8° for PLA/chitin to 66.3° and 65.5° for 5% loading of bamboo CNF (B-CNF) and commercial CNF (C-CNF). The contact angle was reduced with increased CNF loadings from 1 to 5%. The lowest contact angle was obtained at 5% CNF loading. The bamboo-CNF-reinforced biocomposites had a greater contact angle than the commercial CNF biocomposites, probably due to the higher crystallinity of bamboo CNF compared to commercial CNF [65]. A previous report showed that the reinforcement ability of CNF is dependent on its crystallinity index [18]. This is probably why bamboo CNF had a higher contact angle than commercial CNF. The chief reason for this improvement is the crystallinity of the B-CNF, which maintains a higher contact angle compared to the commercial CNF. The contact angle values showed an increase in the biocomposite surface’s wettability with chitin and CNF [65]. The wettability increase was more significant with the CNF than with the addition of chitin, probably because of the presence of the hydroxide group in chitin and the hydrophilic nature of CNF [18]. The contact angle results also corroborated the results obtained from the FT-IR, which showed the presence of hydroxide (OH) in the biocomposites from both PLA/chitin and PLA/chitin/CNF samples. Generally, whether a biocomposite is still hydrophobic is based on its contact angle values [56]. However, there was an increase in wettability. Wettability is an essential property of polymeric materials intended for packaging applications. The biocomposite’s hydrophobic nature is needed for the water-repelling function of the packaged product [3]. The results of the analysis of the wettability properties of the biocomposites showed that the water repellence of PLA was retained despite the addition of CNF for the enchancement of mechanical strength [18].

## 3. Materials and Methods

### 3.1. Materials

The PLA was obtained from Sigma Aldrich, Pasir Pan-jang Rd, Singapore. The properties of the practical grade PLA 4043D were 53 MPa (tensile strength) and 1.24 (specific gravity). The chitin’s practical grade (90% deacetylated) was purchased from Biobasic, Malaysia. The CNF was prepared from bamboo, and a commercial CNF was obtained from the cellulose lab, Canada, as a standard reference to verify the viability of the isolation technique.

### 3.2. The Isolation and Characterisation of Cellulose Nanofibrillated Fibre from Bamboo

The bamboo CNF was isolated with combined alkaline digestion, chlorine-free pulping, supercritical carbon dioxide defibrillation, acid hydrolysis, and high-pressure homogenisation. The bamboo stalk was cut into small pieces of 20 to 30 mm with a saw and subjected to mild alkaline hydrolysis, using NaOH to obtain the bamboo pulp. The bamboo stalk pieces were heated in 0.2 wt.% of anthraquinone and 25 wt.% alkaline concentration NaOH at 160 °C for 4 h (all percentages were based on the bamboo fibre’s weight) [24]. Chlorine-free bleaching was performd using ozone at 30 °C and a flow rate of 0.5 L/min to obtain bleached fibre from the bamboo. The bleached fibre was washed in distilled water to remove excess chemicals. The bleached fibre bundle was defibrillated into microsize using supercritical carbon dioxide explosion at a pressure of 50 MPa for 2 h at a temperature of 60 °C. This was performed in order to loosen the fibres to enhance the fibre surface area with acid during hydrolysis. After that, the defibrillated fibres were subjected to mild acid hydrolysis using oxalic acid (0.2 M at 40 °C) to obtain cellulose microfibrillated fibre. The isolated microfibrillated fibre was homogenised at 56 MPa pressure at 44 homogenisation cycles in order to obtain cellulose nanofibrillated fibre. The transmission electron microscope image of the isolated CNF was obtained using a TEM machine from Perkin-Elmer, PC1600, Winter Street Waltham, MA, USA at 40 kV and with a 100 nm scale size. The CNF sample was stained with acetone on a sensitive copper gauze, and the image was taken under the TEM machine. The fibre size was confirmed with a particle suze analyser from Zetasizer Ver. 6.11, Malvern, UK. The CNF FT-IR functional group analysis was performed in order to verify the structural properties. The FT-IR was performed with a FT-IR EFTEM Libra from Carl Zeiss, Selangor, Malaysia, using film produced by mixing the powdered CNF with KBR. The pressed film absorbance band was obtained for both types of CNF. Furthermore, the X-ray diffraction (XRD) analysis of the CNF was obtained using PANalytical X’Pert PRO X-ray Diffraction (Malvern Panalytical, Techlink, Singapore) at 45 volts, with a 40 A tube current, 1.540598 for K-alpha 1 and K-alpha 2, and a wavelength 2θ = 10° to 70°. The zeta potential colloidal stability of the isolated CNF suspension in water was analysed using Zetasizer Ver. 6.11 (Malvern, UK). A similar test was conducted for the commercial CNF. The TGA-DTA was measured using a PerkinElmer TG-IR-GCMS Interface Q500, TA Instruments (PerkinElmer Inc., Akron, OH, USA), for 5 to 10 mg of the CNFs, at 20 °C/min and a temperature range of 40 to 800 °C

### 3.3. The Preparation and Characterisation of CNF-Reinforced Bionanocomposite

Polylactic acid was blended with chitin at a percentage ratio of 90:10 to form the matrix, using a rheomixer [39]. The blend was then reinforced with 1%, 3%, and 5% cellulose nanofibre [18]. The polymer mix was extruded in a twin-screw extruder Process 11 extruder from Thermo Scientific (Waltham, MA, USA), at a temperature profile of 120 to 180 °C and a feeding rate of 100 g/min, to obtain PLA/chitin/CNF biocomposite. The filament was pelletised with a Varicut Pelletizer 11 M (Thermo Fisher Scientific, Waltham, MA, USA). The pellets were spread in a rectangular stainless-steel mould and pressed with a Carver Press (model 3851-0) (Carver, Wabash, IN, USA) into a biocomposite board at 170 °C for 15 min. The board was cut into test samples for physical, mechanical, thermal, and wettability analysis.

The physical properties of neat PLA, PLA/chitin, and PLA/chitin/CNF were studied for moisture content determination, water absorption, swelling thickness, and moisture content measurement. The percentage of water absorption was measured by pre-weighing 2 cm × 2 cm cut samples and immersing them in 500 mL distilled water. The samples’ final weight was taken after 24 h according to ASTM D570-98. The weight was measured using a jaw-type Mitutoyo digital Vernier calliper with an accuracy of 0.01 mm. The water absorption properties were determined using Equation (1):

(1) $$\mathrm{Water}\;\mathrm{absorption}\;\left(\%\right)=\frac{W_{2}-W_{1}}{W_{1}}\times 100$$

where W_1_ and W_2_ are the initial and final weights of the samples.

The thickness of the biocomposite’s swelling was calculated by measuring the initial and final thickness of the water absorption samples using a micrometre screw gauge. The value of the percentage thickness of swelling was calculated with Equation (2):

(2) $$\mathrm{Thickness}\;\mathrm{of}\;\mathrm{swelling}\;\left(\%\right)=\frac{t_{2}-t_{1}}{t_{1}}\times 100$$

where t_1_ and t_2_ are the initial and final thickness of the samples.

The moisture content of the biopolymer composite was determined using the ASTM D6980-7 standard. PLA/chitin/CNF biocomposite was cut into 2 cm × 2 cm and preweighed before being oven-dried at 60 °C until a constant weight was achieved. The value of the final weight was obtained, and the moisture content was determined using Equation (3):

(3) $$\mathrm{Moisture}\;\mathrm{content}\;\left(\%\right)=\frac{W_{1}-W_{2}}{W_{1}}\times 100$$

where W_1_ and W_2_ are the initial and final weights of the samples.

The biocomposite’s density was determined by measuring the mass (m) and thickness (t) of the 2 cm × 2 cm cut samples. The density was calculated using Equation (4):

(4) $$\mathrm{Density}=\frac{m}{V}$$

where V = l × b × t is the volume of the cut samples.

The mechanical properties of the neat PLA, PLA/chitin, and PLA/chitin/CNF biocomposites were measured with tensile properties, flexural properties, and impact strength determination. The tensile strength, Young’s modulus, and elongation measurements of the biocomposites were obtained from the tensile test analysis that was performed using an MT1175 (Dia-Stron Instruments, Andover, UK) machine at ASTM 638. The dumbbell-shaped samples, whose dimensions were 165 mm × 19 mm × 3 mm, were positioned in the machine at a tensile force of 50 kN and a speed of 60 mm/min. The tensile test was conducted for five replicates of each sample, and the average values with the standard error were recorded.

The flexural test was conducted using ASTM D790 standard polymer composite testing. The samples were cut into rectangles of 200 mm × 12.7 mm × 3 mm and placed in the Instron universal testing MT1175 (Dia-Stron Instruments, Andover, UK) machine. A machine load capacity of 50 KN was applied at a rate of 2 mm/min. The flexural strength and modulus were obtained and analysed for neat PLA and biocomposites.

The impact properties of neat PLA, PLA/chitin, and PLA/chitin/CNF biocomposites were tested using the Izod notched impact method, according to ASTM-D256. The samples were cut to 70 mm × 15 mm × 4 mm and notched at the centre (V-shaped) and placed in an impact tester machine (Model: Gotech GT-A1-7000L). The samples were tested in replicates of five for each composition.

The morphological and structural properties of the biocomposites were determined with SEM, XRD, and FT-IR analysis. SEM micrographs were obtained using EVO MA 10, Carl-ZEISS SMT, Oberkochen, Germany, and the low-magnification morphological images for each sample were captured. The FT-IR analysis of the biocomposites was performed in order to study the formation of bonds in the biocomposites obtained using an FT-IR EFTEM Libra (Carl Zeiss, Selangor, Malaysia). The biocomposite powdered form was mixed with potassium bromide (KBr) and pressed into the circular film. The film absorption band was obtained using the FT-IR machine. The XRD studies were conducted in a similar way to those of the CNF samples. The thermogravimetry analysis of the biocomposites was performed in order to obtain their thermal degradation properties. The TGA-DTA was measured using a PerkinElmer TG-IR-GCMS Interface Q500, TA Instruments (PerkinElmer Inc., Akron, OH, USA), for 5 to 10 mg of the biocomposite, at 20 °C/min, and in a temperature range of 40 to 800 °C. The weight loss and derivative weight loss per minute per temperature change were obtained and plotted. The wettability properties of the biocomposites were studied using a water contact angle. The biocomposites were cut to square size (2 cm × 2 cm) and placed in the machine. The drop of water was observed on the surface of the prepared sample using a KSV CAM 10 (KSV Instruments Ltd., Espoo, Finland) machine. Five replicates of each sample were tested, and the average contact angle, with its standard deviation, was recorded.

## 4. Conclusions

Cellulose nanofibre was successfully isolated from bamboo using a combined supercritical carbon dioxide pre-treatment, hydrolysis, and high-pressure homogenisation method. Compared with commercial CNF, the characterisation results proved that the preparation method and sources affect the fibre size and crystallinity index. The TEM images showed a network of fibres of diameters ranging from 10 to 18 nm. The FT-IR functional group analysis proved the successful isolation of cellulose nanofibre from bamboo by removing the lignin and non-cellulosic functional group from the bamboo fibre. The comparative properties of bamboo CNF and commercial CNF showed that the reinforcement ability of CNF is dependent on its source and method of isolation. The result showed that the source directly affects the crystallinity index, while the production method determines the fibre size distribution of the isolated CNF. The properties of the biocomposites produced from B-CNF and C-CNF were observed to be significantly different. This was due to the differences between the crystallinity and fibre size properties of the B-CNF and C-CNF. The tensile, flexural, and impact properties of the B-CNF-reinforced biocomposites were higher than those of the C-CNF-reinforced biocomposites. Furthermore, the thermal properties suggested that the bamboo CNF shows better thermal stability properties, according to the TGA-DTG analysis. However, the physical properties, such as water absorption, swelling thickness, and moisture content of B-CNF were lower than those of C-CNF, which was probably due to the barrier caused by the higher crystallinity index of B-CNF. All fabricated biocomposites showed good mechanical, wettability, thermal, and morphological properties, which makes them suitable for several material applications. This study provides a simple method of preparing biocomposites with standard industrial techniques. The technique and properties used can be adapted to the large-scale production of biocomposites for industrial applications.

## Figures and Tables

**Table 1:** Contact angles of bamboo and commercial CNF biocomposites.

Filler Contents (wt.%)	Droplet Image	Contact Angle for PLA/Chitin Embedded with B-CNF (θ)	Droplet Image	Contact Angle for PLA/Chitin Embedded with C-CNF (θ)
P90/10	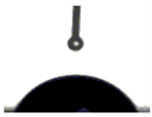	78.80° ± 0.23 ^d^ 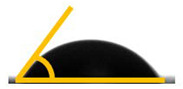		
90/10/1	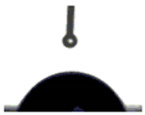	74.24° ± 0.18 ^d^ 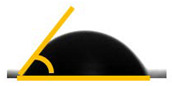	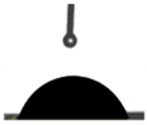	73.70° ± 0.22 ^de^ 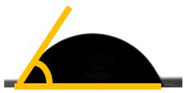
90/10/3	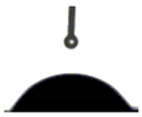	70.80° ± 0.27 ^c^ 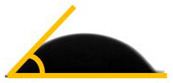	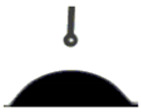	69.80° ± 0.24 ^c^ 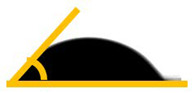
90/10/5	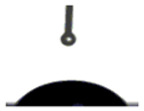	66.80° ± 0.27 ^a^ 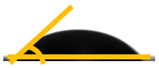	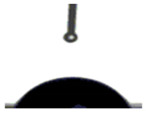	65.80° ± 0.20 ^b^ 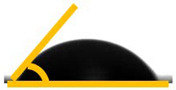

Mean values with the different superscript a–e indicates significant different *p* > 0.05.

## Data Availability

Not applicable.

## Sample Availability

Samples of the biocomposites are available from the authors.
